# Mapping differentiated cognitive landscapes of university students in China via fuzzy comprehensive evaluation

**DOI:** 10.3389/fpsyg.2026.1740777

**Published:** 2026-02-24

**Authors:** Mei Liu, Yaolong Lu, Kai Zhang, Li Xia

**Affiliations:** 1 Institute of Higher Education, Anhui University, Hefei, Anhui, China; 2 School of Mathematical Sciences, Anhui University, Hefei, Anhui, China; 3 School of Education, Hefei University, Hefei, Anhui, China

**Keywords:** cognitive landscapes, cognitive-emotional-behavioral framework, Confucian-heritage culture, fuzzy comprehensive evaluation, academic failure

## Abstract

**Introduction:**

This study investigates the complex underlying factors contributing to academic failure among students at elite Chinese universities. Grounded in cognitive-behavioral theory, we developed a three-dimensional framework encompassing 26 influencing factors across cognitive, emotional, and behavioral domains.

**Methods:**

Utilizing the Fuzzy Comprehensive Evaluation methods ( N=722 ), we identified distinct cognitive maps for three academic performance groups: high-achieving, academic fluctuating, and academic struggling.

**Results:**

Findings reveal that academic failure is characterized by a “cognitive detachment” rather than mere knowledge deficiency or emotional indifference. Specifically, (1) high-achievers demonstrate a “productive tension,” where negative psychological attributes like self-doubt and learning anxiety function as significant positive drivers within the Confucian-heritage cultural context; (2) Fluctuating students exhibit high environmental sensitivity, where academic stability is contingent upon perceived support from family and peer networks; (3) Struggling students acknowledge their academic deficiencies but appear cognitively detached from the emotional impact of failure, aligning with a “lying flat” mentality fostered by current institutional evaluation models.

**Discussion:**

Theoretically, this research moves beyond linear “cause-effect” narratives to uncover how specific cognitive-emotional configurations shape academic trajectories. Practically, it highlights a need for differentiated university interventions that move beyond standardized counseling toward rebuilding “agency-performance” links, while inviting a reflective reconsideration of the core purpose of university examination systems.

## 1 Introduction

Understanding the factors that influence college students’ academic performance is a priority for university administrators (van Rooij et al., 2018; Molavi, 2024). While scholarship has identified numerous individual and contextual influencers (Honicke and Broadbent, 2016; Salloum et al., 2017), a paradoxical gap remains: why do some students who demonstrated exceptional prowess in high-stakes exams – specifically China’s GaoKao (Wu et al., 2020) – frequently encounter significant underperformance or course failure after enrollment? This contradiction suggests that the underlying mechanisms governing how students internalize influencing factors remain poorly understood, as prior research often relies on simplified linear input–output models that fail to account for the psychological complexity of student transitions. Consequently, while researchers and administrators have identified numerous factors and developed various intervention measures – such as strengthening social support system (Shan et al., 2022) and implementing individualized learning interventions (Zhang et al., 2019) – the efficacy of these measures remains subject to doubt. This gap highlights a critical disconnect between broad intervention strategies and the nuanced, differentiated cognitive landscapes of the students themselves.

In response to these conceptual limitations, this study employs fuzzy logic not merely as a technical tool, but as a framework to capture the inherent ambiguity and non-linear interactions within student self-perceptions. By surveying 960 students from a Chinese “Double First-Class” university, we focus on their authentic self-evaluations across 26 factors. The scope of this research is specifically bounded by these self-perceived influencers rather than objective determinants, aiming primarily to classify divergent academic trajectories – high-achieving, fluctuating, and struggling – and explain their unique cognitive-emotional landscapes. Theoretically, we delineate the cognitive disparities that shape these trajectories, constructing distinct cognitive maps; practically, we provide a basis for universities to design targeted interventions that move beyond standardized metrics to support the unique needs of at-risk students.

## 2 Theoretical framework

As previously noted, extensive research has identified numerous factors influencing academic performance. However, the interrelationships among these factors remain intricate. Take self-efficacy as an example. Honicke and Broadbent (2016), through a systematic review of 59 relevant studies, found that self-efficacy not only has a direct causal relationship with academic performance but also involves other mediating and moderating factors (such as self-regulation and processing strategies). Precisely because of these complex interactions, the explanatory power of existing factors influencing academic performance is constrained. This study does *not* aim to identity new factors affecting academic performance, *nor* does it intend to elucidate the relationships between different influencing factors. Instead, it seeks to explore the contribution of factors that have been empirically validated to influence academic performance (including both direct and indirect factors) to differences in academic performance – specifically, whether the influence of these factors varies among students with distinct performance levels (i.e., high-achieving, fluctuating, and struggling).

**Figure 1:**
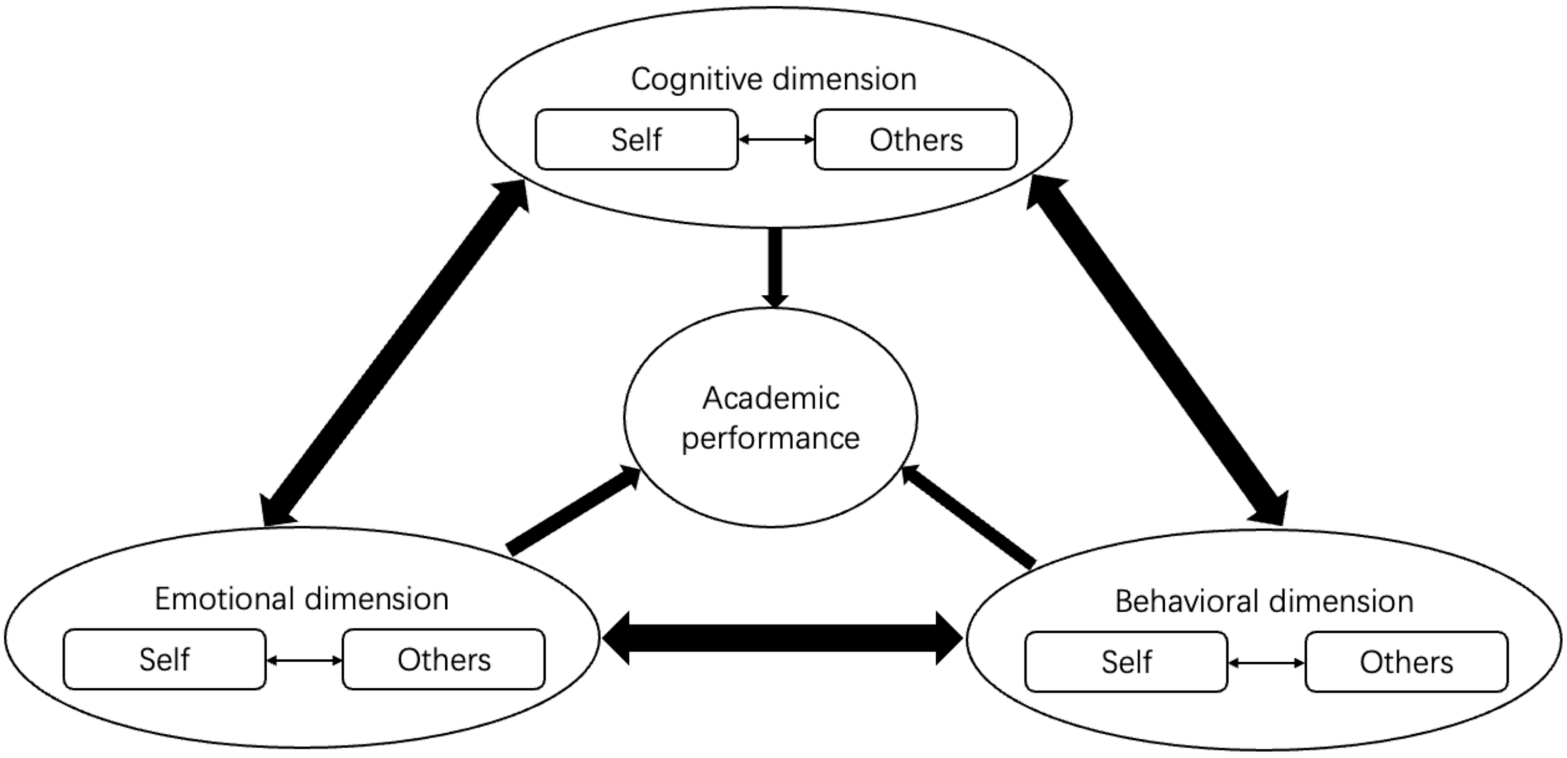
Theoretical framework of this study.

While these students admitted via the Gaokao are unlikely to have learning disabilities, their academic outcomes are viewed here through the lens of adaptive behavioral choices. However, we justify this focus by acknowledging that such “choices” are not made in a vacuum but are bounded by structural and institutional constraints, such as curriculum rigidity or varying access to academic resources (Hanushek and Woessmann, 2014). Grounded in Cognitive-Behavioral Theory (short as CBT below), we posit that academic failure reflects a student’s active withdrawal from learning behaviors as a psychological response to their perceived environment. Their responses are driven by three core dimensions: cognition, emotion and behavior (Suldo et al., 2008), each of these dimensions further comprises two primary aspects: orientation toward the self and orientation toward others (Nurius and Macy, 2012). Crucially, self-oriented and other-oriented factors within these dimensions are not parallel silos but functionally interdependent. For instance, a student’s internal cognitive efficacy is often continuously recalibrated by other-oriented emotional feedback, such as perceived parental expectations or peer comparisons. Our framework captures these reciprocal interactions, viewing them as the mechanism that determines the final behavioral output (see Figure 1). We, therefore, propose that the factors influencing college students’ academic failure can be systematically summarized within this framework.

### 2.1 Cognitive dimension

Self-efficacy – defined as an individuals’ belief in their capacity to execute behaviors necessary to achieve specific performance outcomes (Lane et al., 2004) – serves as a foundational component of the cognitive dimension. Beyond self-efficacy, studies have confirmed other self-related influencing factors, including perceptions of own professional knowledge (Smeby, 2007), awareness of personal academic strengths (Saldaña et al., 2014), and cognitive orientations toward planning and prioritizing tasks (Greiner and Karoly, 1976).

In the cognitive dimension, there also exist influencing factors related to external others. Foremost among these is the direct impact of family-related factors (e.g., perceptions of family socioeconomic status and family relationship stability) on academic performance (Sothan, 2019). Also, Yamamoto and Holloway (2010) demonstrated that in Asian cultural contexts, students’ cognitive interpretations of their parents’ academic expectations significantly shape their own academic goals and effort. Besides family-related factors, institutional and social cognitive factors also exert influence on academic performance. For example, Nzoka and Orodho (2014) highlighted that perceptions of university management styles shape students’ sense of belonging and institutional trust; similarly, Gallardo et al. (2016) found that peer relationship quality – grounded in cognitive assessments of social support and collaboration – directly impacts academic engagement and performance. Lastly, and equally importantly, academic outcome expectations (Levi et al., 2014) are also categorized under “other aspects” in this study.

In summary, the cognitive dimension acts as the “interpretive lens” through which students process their academic reality. It integrates internal beliefs of capacity (such as self-efficacy) with external structural perceptions (such as family status), forming a holistic mental representation that guides task prioritization and goal setting.

### 2.2 Emotional dimension

Learning interest serving as an intrinsic motivator for engagement, elicits positive affective experiences in students. These positive emotions not only enhance immediate learning enjoyment but also strengthen long-term motivation, thereby indirectly boosting academic performance (Urhahne and Wijnia, 2023). Closely tied to motivation is the sense of academic accomplishment - a situational, state-like affective-cognitive construct arising from the successful completion of learning goals or tasks (Schunk and Zimmerman, 2007). Additionally, Chong and Reinders (2025) conclude that higher levels of learning autonomy correlate with improved academic performance. Besides, the influence of some negative emotions (such as anxiety and self-doubt) remain contested in academic research: while most scholars argue that higher levels of these negative emotions are associated with poorer academic performance (e.g., Woodman et al., 2010), Stankov (2010) found that in Confucian cultural contexts, academic performance and self-doubt and learning anxiety exhibit a positive correlation – meaning higher self-doubt and anxiety levels are linked to better academic outcomes.

As for other-related emotional factors, their emotional targets primarily orient toward external elements, encompassing emotional experiences (such as fear) related to course failure (Nsiah, 2017), emotional attitudes toward college life and learning (including the quality of course content, teaching quality, and assessment methods) (Bailey and Phillips, 2016), and emotional responses regarding future employment (Saha and Sikora, 2008). All these factors can either contribute to students’ academic success or lead to their course failure in the university context. As summarized by Pekrun et al. (2017), positive emotions were positively correlated with performance, whereas negative emotions were negatively correlated with performance.

In summary, the emotional dimension within this framework functions as the “affective engine” that modulates how students internalize their academic environment. While Pekrun et al.’s (2017) assumptions generally posits a linear correlation where positive emotions facilitate performance and negative emotions hinder it, this relationship requires theoretical recalibration within the Confucian cultural context. In high-stakes, competitive academic environments like China’s, the traditional dichotomy between “positive” and “negative” valence may be blurred (Hofstede et al., 2010). For instance, emotions such as self-doubt or performance-related anxiety – typically viewed as debilitating in Western psychological models – can theoretically function as pro-social motivators tied to familial expectations and social unacceptability of failure (Stankov, 2010). By incorporating these culturally situated affective-cognitive constructs, the proposed framework allows for a more nuanced mapping of emotional landscapes that can account for non-linear “paradoxes” in student motivation.

### 2.3 Behavioral dimension

The behavioral dimension also encompasses self-oriented and other oriented influencing factors. For instance, self-regulation, sustained learning attention, stress resilience, and reflective ability are all categorized as self-oriented behavioral factors, could impact on college students’ learning outcomes (Donker et al., 2014). Similarly, the application of learning strategies and methods, peer and social management skills, along with the mastery of basic learning skills, fall under other-oriented behavioral factors (Pintrich and Zusho, 2007).

In summary, the behavioral dimension serves as the critical “manifestation layer” where internal cognitive-emotional states are translated into observable academic actions. This dimension synthesizes self-oriented regulatory behaviors – such as sustained learning attention, stress resilience, and reflective ability – with other-oriented competencies, including the mastery of learning methods and social management. For a theoretical perspective, while these behaviors appear as individual “choices,” they are deeply situated within the “strict-entry-and-lenient-exit” institutional framework of Chinese higher education (Zhang et al., 2025). In this context, behavioral output is not merely an autonomous decision but an adaptive response to the interaction between a student’s self-regulatory strength and their mastery of social-academic navigation strategies. Furthermore, consistent with the cultural specificity of Confucian-heritage systems, “effective” learning behavior often entails a sense of moral duty toward familial expectations, which may paradoxically sustain academic effort even amidst emotional turbulence.

Based on the preceding summary and synthesis, this study has identified a total of 26 specific influencing factors, which are categorized into two aspects (self-related and other-related) within each of the three overarching dimensions (list of the factors could be found in Table 1). All these influencing factors have been validated by prior research, demonstrating their positive, negative, direct, or indirect effects on academic performance.

**Table 1:** The description results of each factor.

Dimension	Aspect	Indicator	Mean	SD	Skewness	Kurtosis
Cognitive dimension	Self	Self-Efficacy	5.68	2.03	−0.25	−0.11
		Perceptions of own professional knowledge	5.40	1.85	−0.12	0.14
		Awareness of personal academic strengths	5.56	1.87	−0.20	−0.03
		Cognitive orientations toward planning and prioritizing task	5.95	1.88	−0.30	−0.07
	Others	Perceptions of family socioeconomic status	5.78	1.75	−0.34	0.33
		Family relationship stability	7.08	2.06	−0.46	−0.25
		Parents’ academic expectations	6.66	1.97	−0.57	0.31
		Perceptions of university management styles	5.81	2.01	−0.46	0.01
		Peer relationship quality	6.69	1.94	−0.52	0.31
		Academic outcome expectations	5.37	2.16	−0.12	−0.52
Emotional dimension	Self	Learning interest	5.75	2.17	−0.38	−0.42
		Sense of academic achievement	5.73	2.10	−0.31	−0.21
		Learning anxiety	6.34	2.15	−0.32	−0.33
		Learning autonomy	6.47	2.10	−0.63	0.10
		Self-Doubt	5.41	2.00	−0.02	−0.11
	Others	Emotional experiences related to course failure	6.28	2.88	−0.45	−0.83
		Emotional attitudes toward quality of course content	6.16	1.79	−0.31	0.37
		Emotional attitudes toward teaching quality	6.24	1.94	−0.44	0.15
		Emotional attitudes toward assessment methods	5.81	2.01	−0.45	−0.02
		Emotional responses regarding future employment	5.73	2.24	−0.34	−0.44
Behavioral dimension	Self	Self-regulation	6.41	2.08	−0.48	0.10
		Sustained learning attention	5.62	1.82	−0.11	0.11
		Stress resilience	6.30	2.01	−0.36	0.15
	Others	Ability of learning strategies and methods	5.96	1.67	−0.28	0.48
		Peer and social management skills	5.87	2.15	−0.37	−0.14
		Mastery of basic learning skills	5.98	1.83	−0.45	0.40

As mentioned above, this study aims to explore whether different influencing factors exert heterogeneous effects on students with varying academic performance. Taking self-efficacy as an example, although prior research has indicated that higher self-efficacy is associated with better academic performance, a critical question remains: do high-performing students attach greater importance to self-efficacy, while low-performing students value this factor less? Answering this question will enable systematic mapping of the cognitive landscapes of students with varying academic performances, thereby elucidating the paradox introduced at the outset of this study – namely, why students who achieved “success” in Gaokao frequently experience academic failure in university examinations after enrollment.

## 3 Methods

Drawing on the approach of fuzzy comprehensive evaluation, we developed a questionnaire covering the above 26 factors and administrated it to 960 students at a High Level university in China. Next, we will first provide a detailed account of the questionnaire development process and the application of the Fuzzy Comprehensive Evaluation method (short as FCE) in this research. Subsequently, we will present the research context and data collection procedures.

### 3.1 Questionnaire development process

After introducing the research purpose and confidentiality measures to participants at the beginning of the questionnaire, we asked them to self-report their number of failed courses, with options: *None*, *1–3 courses*, or *4 or more courses*. Additionally, to examine college students’ self-evaluation of these factors, we asked students to assess their own levels of each factor. For example, regarding self-efficacy, based on its definition, the item was phrased as: “*When facing challenging academic tasks, my level of confidence is:*.” For learning interest, the item was “*The extent of my interest in my major is:*.” For stress resilience, the item was “*My ability to cope with stress is:*.” Each of the 26 items corresponds to one of the 26 influencing factors.

While some constructs in this study, such as self-efficacy and learning autonomy, are multi-faceted, we employed single-item self-report measures for each of the 26 factors to reduce participants fatigue and ensure a high completion rate among the 960 respondents. Although formal reliability (e.g., Cronbach’s alpha) and validity testing are typically required for multi-item scales, the use of single items is justified here by the face validity (Nevo, 1985) established through group interviews with 15 students, who confirmed that each item clearly and comprehensively captured the intended construct in the context of their daily academic lives. Future studies may benefit from expanded multi-item scales to further validate these findings.

### 3.2 The application of the fuzzy comprehensive evaluation method

Before introducing the application of the FCE method, it is essential to first clarify its core nature — what kind of method it entails — and the rationale for selecting this approach in the current study.

FCE is a specialized data processing method grounded in the theoretical framework of Fuzzy Logic (FL). Initially proposed by Zadeh (1965), FL enables the description of system behavior through an approximate yet effective approach, rather than relying on traditional linear functions, thereby facilitating successful system modeling (Zadeh, 1965). Its core principle lies in the assertion that “everything is a matter of degree” (Gravani et al., 2007). Intuitively, FCE can be understood as a method that “mathematizes” subjective human judgment. Unlike traditional statistics that force a binary “yes or no” (e.g., a student is either motivated or not), FCE recognizes that internal perceptions exist in degrees of truth. By transforming these qualitative degrees into a quantitative evaluation matrix, FCE allows us to synthesize multiple fuzzy opinions into a single rigorous evaluation score for each performance group. In this study, the relationships between the 26 influencing factors and academic performance are extremely complex, making linear models nearly inadequate for describing them. However, since the primary objective of this research is to distinguish differences in the degree of influence among various factors, we, therefore, draw on the principles and methods of FL, i.e., FCE.

The application of FCE in this study unfolds in five core steps:

#### 3.2.1 Defining the linguistic evaluation set

Given that the questionnaire requires participants to rate their performance, abilities and satisfaction across the target influencing factors, this study established a 5-level linguistic evaluation set (see Table 2).

**Table 2:** Correspondence table: ten-point scale, descriptive comments, and quantitative assignments.

10-Point Scale	1–2.8	2.8–4.6	4.6–6.4	6.4–8.2	8.2–10
Rating Set	Very Low/Very Poor/Very Dissatisfied	Low/Poor /Dissatisfied	Moderate/Neutral	High/Good/Satisfied	Very High/Excellent/Very Satisfied
Quntitative Assignment (*V*)	*V*_2_=2	*V*_3_=3	*V*_4_=4	*V*_4_=4	*V*_5_=5

#### 3.2.2 Constructing membership functions

To improve the precision of self-ratings, the questionnaire adopted a 10-point evaluation, allowing participants to select a range within this scale (e.g., 6–8). The correspondence between the 5 fuzzy levels (from Step 1) and the 10-point scale is detailed in Table 2.

The entropy method was employed to process the data for each factor. First, Equation 1 calculated the proportion of the *j*-th evaluation factor’s data for the *i-*th sample:


$$p_{\mathrm{ij}}=\frac{x_{\mathrm{ij}}}{\sum_{i=1}^{n}x_{\mathrm{ij}}}$$ (1)

Where $x_{\mathrm{ij}}$ denotes the raw data of the *i-*th sample for the *j*-th factor. Next, Equation 2 computed the entropy $e_{j}$ of the *j*-th factor, quantifying the dispersion of its data:


$$e_{j}=-\frac{1}{\ln(n)}\sum_{i=1}^{n}p_{\mathrm{ij}}\ln(p_{\mathrm{ij}})$$ (2)

A smaller $e_{j}$ indicates greater data dispersion, more information provided by the factor, a more significant role in comprehensive evaluation, and consequently, a larger weight. Finally, Equation 3 determined the weight $\omega_{j}$ of the *j*-th factor.


$$\omega_{j}=\frac{1-e_{j}}{\sum_{j=1}^{m}(1-e_{j})}$$ (3)

where n is the total number of samples and m is the total number of factors.

#### 3.2.3 Constructing the fuzzy matrix

Combining academic achievement influence factors and their corresponding evaluation data, the membership degree coefficients for each factor were calculated, and a fuzzy relational matrix R for different student groups of academic influence factors was constructed. For the *j*-th factor in the factor set, if its membership degree to the first level of the evaluation set V is $r_{j1},$ the evaluation result of the j -th influence factor was represented as a fuzzy set:


$$R_{j}=(r_{j1},,,,r_{j2},,,,r_{j3},,,,r_{j4},,,,r_{j5})$$ (4)

Based on Equation 4, a 5 × 26 fuzzy relational matrix R was constructed, summarizing the fuzzy evaluation of 26 academic influence factors across 3 student groups (i.e., high-achieving, academic fluctuating group and academic struggling group). This study employed the fuzzy statistical method; based on sample survey evaluation results, membership degrees were defined using membership frequencies.

#### 3.2.4 Calculating comprehensive evaluation value (CEV) and weights value (WV) of each factor

To interpret the findings accurately, a distinction must be made between Comprehensive Evaluation Values (CEV) and Weighting Values (WV). The CEV reflects the perceived status of each factor – how students evaluate their own standing (e.g., their level of self-efficacy). In contrast, the WV represents the degree of impact or the relative importance of that factor in differentiating one performance group from anther. By synthesizing these two metrics, we can identify which factors student lack (low CEV) and which of those deficiencies are most critical to their academic trajectory (high WV).

The CEV of each factor was calculated using Equation 5:


$$F=R\cdot V^{T}$$ (5)

where V is the quantitative assignment vector of the linguistic levels ( $V={\left[V_{1},,,,V_{2},,,,V_{3},,,,V_{4},,,,V_{5}\right]}^{T}$ ). The CEV reflected the overall evaluation score of a sample (e.g., a student group) for a specific factor, indicating how well the factor’s performance aligned with the evaluation criteria.

The WV was calculated using Equation 6 as the product of a factor’s weight ( ω ) and its CEV ( F ):


WV=ω⋅F (6)

This value integrates both the factor’s importance (weight) and its performance (CEV).

#### 3.2.5 Comparing the results of CEV and WV

This study compared the CEV and WV across different academic performance groups. Specifically, a higher CEV indicated a higher overall evaluation of the factor by the group. And a higher WV signified a greater level of emphasis placed on the factor by the group. In our model, the Entropy Weighting Method was utilized to objectively calculated factor weights based on the dispersion of student self-ratings, effectively reducing the influence of subjective bias in the weighting process. To identify significant disparities between groups, a 1.2-fold criterion was adopted (consistent with Pang et al., 2025). This threshold was selected as a conservative yet sensitive measure to highlight factors where the influence level in one group markedly deviates from the baseline of others, ensuring that the identified “cognitive maps” reflect substantial rather than marginal perceptual shifts.

### 3.3 Research context and data collection

This study selected a High-Level university (hereinafter referred to as University A) as the research setting, with site selection guided by two core principles: (1) Reasonable Academic Stratification. University A has an established dynamic academic early-warning mechanism: students accumulating 10–15 failed credits (about 3 courses) trigger a “yellow warning,” while those with >15 failed credits initiate a “red warning”. Following warnings, interventions (e.g., parent notification, student meetings) are implemented to ensure students recognize their academic standing. Due to these academic warnings and intervention measures, students at University A are assumed to be sensitive to course failure. This assumption is grounded in Institutional Pressure Theory (Zucker, 1987), suggesting that clear behavioral consequences (e.g., parent notification) heighten students’ awareness of their academic standing. Theoretically, this sensitivity aligns with our hypothesis: that academic outcomes are results of self-selection: when faced with explicit failure thresholds, a student’s continued withdrawal from learning behaviors represents a conscious psychological positioning rather than a lack of information. (2) Convenient Data Collection. The authors have extensive familiarity with University A’s undergraduate academic quality. Additionally, this study selected third-year undergraduate students from University A as research participants, primarily because students at this stage have generally formed consistent understanding of academic requirements, campus environments, and their own developmental orientations, enabling them to provide more accurate feedback on their genuine academic states.

Formal data collection then proceeded via paper questionnaire distribution, coordinated by class tutors who liaised with class monitors. A total of 960 questionnaire was distributed, yielding 788 retrieved responses. After excluding invalid samples – including those with incomplete answers and those selecting the same option for 10 consecutive items – 722 valid samples remained, corresponding to an effective response rate of 75.2%. Demographic characteristics of the sample are presented in Table 3.

**Table 3:** Sample demographics.

Type	Category	Frequency
High-achieving Group	None	589
Academic Fluctuating Group	1–3 courses	99
Academic Struggling Group	4 or more courses	34
/	Total	722

### 3.4 Analysis

Prior to the formal application of the FCE method, a comprehensive descriptive statistical analysis was conducted on the 26 factors ( N=722) . Notably, unlike traditional psychometric approaches, no reverse coding was applied to negative emotional factors (such as learning anxiety or self-doubt). Drawing on the theoretical framework proposed by Stankov (2010), which suggests that within the Confucian-heritage cultural context, certain negative effects can function as academic “drivers” rather than inhibitors, all 26 factors were treated as positive contributors to the evaluation matrix. This decision ensures that the resulting cognitive maps accurately reflect the complex, non-linear relationship between emotional pressure and academic persistence specific to high-level Chinese university students. Following this preliminary assessment, the data were processed according to the five core steps of the FCE method detailed in the preceding section. All weight calculations and fuzzy matrix operations were conducted using MATLAB 2022 software, with all calculated values reported to two decimal places.

## 4 Results

### 4.1 Results of descriptive statistical analysis

The descriptive statistical results for the 26 influencing factors ( N=722) , including Means, Standard Deviation (i.e., SD), Skewness and Kurtosis, are detailed in Table 1.

Overall, the dataset demonstrates excellent psychometric properties for fuzzy modeling, with all skewness and kurtosis values remaining well within the conservative range of ±1 . The universal negative skewness across factors indicates a general positive evaluation bias typical of high-level university cohorts.

In terms of central tendencies, the highest mean scores were observed in environmental factors such as Family Relationship Stability (7.08) and Peer Relationship Quality (6.69), while internal cognitive factors like Academic Outcome Expectations (Mean = 5.37) and Perceptions of Professional Knowledge (Mean = 5.40) received the lowest ratings, indicating a widespread sense of academic uncertainty despite favorable external conditions. Furthermore, the Standard Deviations (SD) ranged from 1.67 to 2.88, with Emotional Experiences related to Course Failure (2.88) and Learning Interest (2.17) exhibiting the highest variability. According to the principles of informational entropy, these high-variance factors carry greater weigh in differentiating students across different academic performance levels. These preliminary findings provide a robust empirical foundation for the subsequent CEV and WV calculation. Finally, the relatively high mean of Learning Anxiety (6.34) supports the theoretical decision to treat negative affects as active drivers in the Chinese educational context, consistent with Stankov’s (2010) assumption.

### 4.2 Result of CEV: self-evaluation differences of factors across students with differing academic performance

The CEV of three types of student groups on each factor could be found in Table 4.

**Table 4:** The CEV of three types of student groups on each factor.

Dimension	Aspect	Indicator	High-achieving Group	Academic Fluctuating Group	Academic Struggling Group
Cognitive dimension	Self	Self-Efficacy	3.12	3.04	2.76
		Perceptions of own professional knowledge	3.04	2.69	2.44
		Awareness of personal academic strengths	3.09	2.89	2.71
		Cognitive orientations toward planning and prioritizing task	3.32	3.01	2.74
	Others	Perceptions of family socioeconomic status	3.17	3.10	3.15
		Family relationship stability	3.80	3.67	3.76
		Parents’ academic expectations	3.64	3.34	3.53
		Perceptions of university management styles	3.14	3.06	3.56
		Peer relationship quality	3.64	3.46	3.26
		Academic outcome expectations	2.97	2.81	3.03
Emotional dimension	Self	Learning interest	3.21	2.84	2.74
		Sense of academic achievement	3.16	2.96	3.03
		Learning anxiety	2.54	2.85	2.68
		Learning autonomy	2.52	2.64	2.26
		Self-Doubt	3.41	3.32	3.26
	Others	Emotional experiences related to course failure	3.41	3.09	3.38
		Emotional attitudes toward quality of course content	3.34	3.31	3.38
		Emotional attitudes toward teaching quality	3.40	3.27	3.74
		Emotional attitudes toward assessment methods	3.15	3.15	3.56
		Emotional responses regarding future employment	3.12	3.01	3.44
Behavioral dimension	Self	Self-regulation	3.46	3.45	3.47
		Sustained learning attention	3.11	2.86	2.91
		Stress resilience	2.94	3.00	2.94
	Others	Ability of learning strategies and methods	3.26	3.11	3.06
		Peer and social management skills	3.24	3.07	2.97
		Mastery of basic learning skills	3.26	3.23	3.12

CEV quantified the perceived status of each factor as reported by students. Before detailing factor-level evaluations, two overarching patterns emerge from Table 4. First, a “perception-performance alignment” is evident for high-achievers, who consistently report high evaluations across most cognitive and behavioral domains. Second, a “satisfaction-engagement paradox” characterizes the struggling group: while they report the lowest evaluations for core competencies like self-efficacy and learning interest, they express the highest satisfaction within institutional elements like teaching quality and assessment methods. This suggests that academic failure in this cohort is intertwined with a psychological detachment from the rigor of university requirements. From Table 4, high-achieving students consistently scored higher in self-evaluations than the other two groups across most influencing factors (16 out of 26), with their ratings predominantly falling at moderate or moderately high levels (i.e., above 3). The fluctuating group exhibited the highest self-evaluations for 3 factors, i.e., learning anxiety, learning autonomy and stress resilience. For the academic struggling group, their self-evaluations on the most factors remained at moderate or moderately low levels (e.g., self-efficacy, perception of own professional knowledge, learning interest, and learning autonomy). However, they expressed the highest satisfaction in their attitudes toward college life and learning (including course content, teaching quality, assessment methods, and future employment).

Beyond the top-performing group, the lowest-performing group is also critical to addressing the research questions of this study. First, among the high-achieving group, the two factors receiving the lowest evaluations (2 out of 26): learning anxiety and emotional attitudes toward assessment methods. Second, among the academic fluctuating group, 12 factors receiving the lowest evaluations, including perceptions of family socioeconomic status, family relationship stability, parents’ academic expectations, perceptions of university management style, academic outcome expectations, emotional experiences related to course failure, course content, teaching quality, and future employment, self-regulation and sustained learning attention. Last, among the struggling group, 12 factors receiving the lowest evaluations, including self-efficacy, perceptions of own professional knowledge, awareness of personal academic strengths, cognitive orientations toward planning and prioritizing task, peer relationship quality, learning interest, learning autonomy, self-doubt, stress resilience, ability of learning strategies and methods, peer and social management skills, and mastery of basic learning skills.

It is noted that the academic struggling group represents a relatively small subset of the total sample ( N=722 ). While this reflects the elite nature of University A where total failure is a minority outcome, the concentration of extreme low evaluations within this small group is statistically significant under the FCE framework. The findings for this group should be viewed as an in-depth mapping of the “at-risk” psychological state, providing critical insights for targeted clinical intervention, even if their numerical frequency is lower than the other two cohorts.

### 4.3 Results of WV: varying degrees of factors’ impact on academic performance

**Figure 2:**
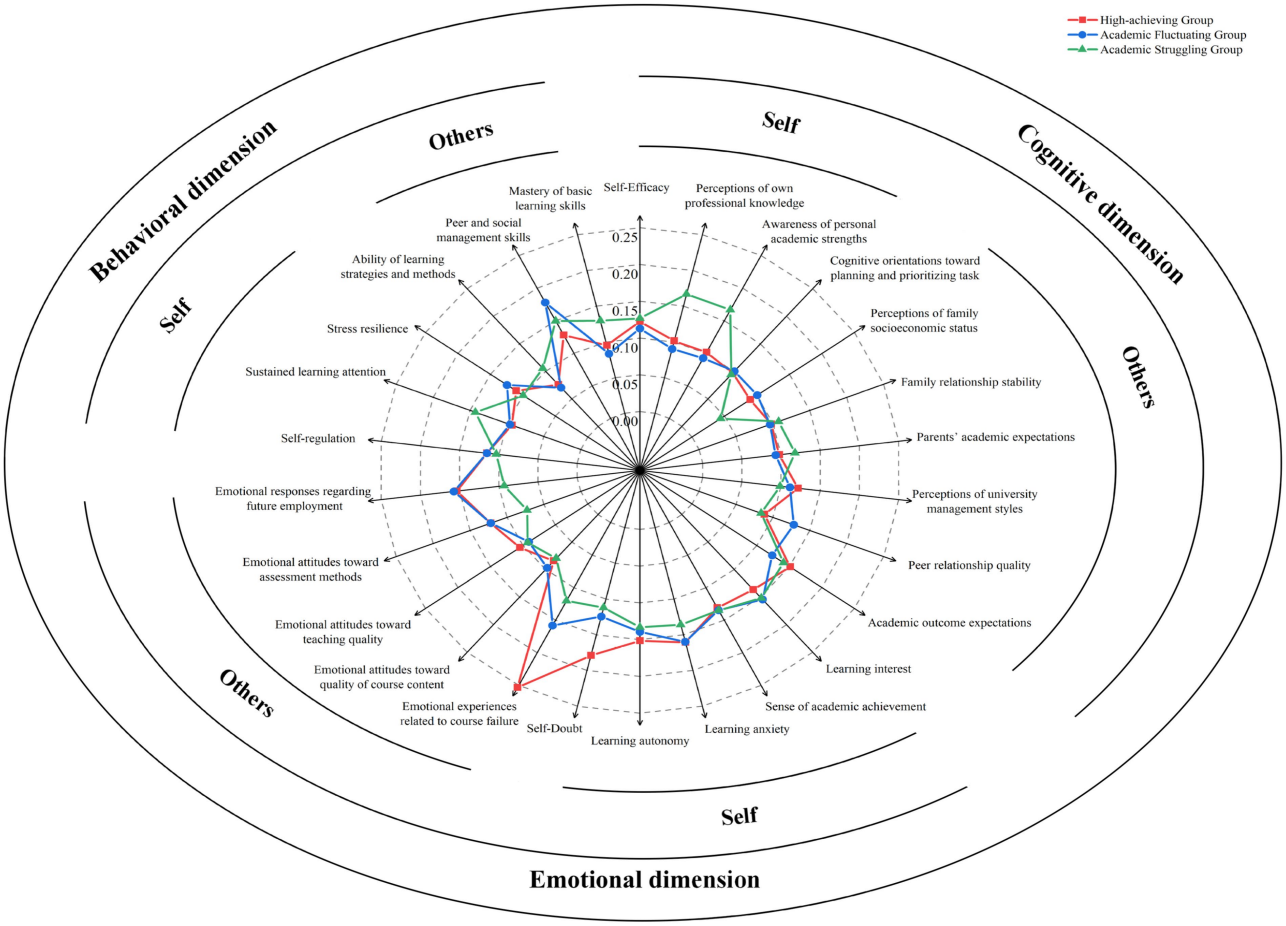
The WV of three student groups across each factor.

The WV of three student groups across each factor could be found in Figure 2.

WV identified the discriminatory power of these factors in shaping academic disparities. As shown in Figure 2, all influencing factors impact the academic performance of every student group — though to varying degrees. Building on the 1.2-fold criterion, the 26 factors are categorized into two groups: (1) Factors that did not significantly affect academic performance disparities (defined as the ratio of the WV of the same factor across different student groups <1.2 ), including 9 factors, self-efficacy, cognitive orientation toward planning and prioritizing task, family relationship stability, learning interest, sense of academic achievement, learning anxiety, learning autonomy, emotional attitudes toward teaching quality, and self-regulation; (2) Factors that significantly contribute to such disparities (ratio ≥1.2 ). These 17 factors with statistically significant differences can be categorized into three types, as outlined below:

Factors significantly impacting the high-achieving group. Ordered by the magnitude of multiples, the ranking is as follows: emotional experiences related to course failure, emotional attitudes toward assessment methods, self-doubt, perceptions of university management styles, and academic outcome expectations.

Factors significantly impacting the academic fluctuating group. Ordered by the magnitude of multiples, the ranking is as follows: perceptions of family socioeconomic status, emotional responses regarding future employment, peer relationship quality, peer and social management skills, stress resilience, and emotional attitudes toward quality of course content.

Factors significantly impacting the academic struggling group. Ordered by the magnitude of multiples, the ranking is as follows: perceptions of own professional knowledge, awareness of personal academic strengths, mastery of basic learning skills, ability of learning strategies and methods, sustained learning attention, and parents’ academic expectations.

## 5 Discussion

This study aimed to explore why university students frequently experience academic exam failures. Grounded in Cognitive Behavioral Theory (CBT), we posit that exam success or failure is a consequence of behavioral choices, shaped by self-related or others-related factors across three dimensions: cognitive, emotional, and behavioral. Building on this hypothesis, we developed an analytical framework encompassing these three dimensions (each with self and others aspects) to systematically categorize the 26 academic performance influencing factors identified in existing literature. Given the complexity of relationships between these factors, we adopted the Fuzzy Comprehensive Evaluation method to develop a *Questionnaire on Factors Influencing Academic Performance* and conducted a survey with 960 students of varying academic performance from a High Level university in China. The results indicate that: (1) Students with different academic performance exhibited differences in their self-evaluation for various influencing factors; (2) The degree of influence of each factor also varies across groups. Based on these disparities, this study extracts cognitive disparities among college students with differing academic performance, thereby enabling a deeper understanding of why students with high college entrance exam scores frequently encounter exam failures after transitioning to university.

### 5.1 Cognitive maps of the three student groups

#### 5.1.1 High-achieving group: productive tension and constructive self-doubt

Our empirical findings reveal a paradoxical cognitive-emotional profile for high-achieving students. According to the WV analysis (Figure 2), emotional experiences related to course failure carry significant weight in their academic performance, while their self-evaluation (CEV) shows notably higher levels of self-blame post-failure compared to other groups. This suggests that for top performers, failure is not merely an academic setback but an intensive emotional catalyst.

Through the lens of sociocultural framework (e.g., Hofstede, 2011), this pattern aligns with competitive atmosphere of Chinese examination culture, where high-stakes outcomes render academic setbacks socially sensitive. Our data indicate that students who internalize these competitive pressures tend to exhibit greater emotional turbulence. Critically, high-achievers also reported higher self-doubt levels (CEV) than their peers, with self-doubt exerting a more pronounced influence (WV) on their success. While traditional Western models might view self-doubt as an inhibitor, these results provide empirical support for Stankov’s (2010) claim that in Confucian-heritage contexts, self-doubt can correlate positively with performance by fostering continuous self-reflection and persistence.

Furthermore, a unique “satisfaction-value paradox” emerged: high-achievers reported the lowest satisfaction with assessment methods yet attached the highest importance (WV) to them. This indicates a strategic adaptation to China’s “strict-entry-and-lenient-exit” university model (Liu et al., 2025). They may not inherently prefer these institutional constraints, but their cognitive maps prioritize them as essential instruments for navigating academic competition and avoiding failure.

#### 5.1.2 Academic fluctuating group: sensitivity to environmental volatility

For the academic fluctuating group, a distinct “high-importance, low-evaluation” contradiction emerged regarding their surrounding environment. Empirical results from the WV analysis (Figure 2) indicate that this group attaches significantly higher importance to external factors – including family dynamics, peer relationships and teaching content – compared to the other two groups. However, their self-evaluations (CEV) for these same factors were consistently among the lowest (Table 4). This suggests that the academic performance of fluctuating students is highly contingent upon external stability; when their perceived environmental support is low; their performance tends to oscillate.

This finding can be tentatively interpreted through the lens of Bourdieu’s theory of social and cultural capital. The perceived deficiencies in familial or social capital may not act as absolute barriers but rather as sources of systemic vulnerability (Jones and Zimmer, 2001; Salloum et al., 2017). Notably, our data show that family-related factors (e.g., socioeconomic status and parental expectations) exert a particularly pronounced influence (WV) on this group, while their average CEV scores remained significantly below those of high-achievers. Unlike the struggling group, who appear more detached from these metrics, the fluctuating group remains deeply invested in these external capitals, rendering their academic outcomes susceptible to perceived environmental inadequacies.

#### 5.1.3 Academic struggling group: cognitive detachment and the “lying flat” logic

For students in the academic struggling group, a notable asynchrony between perceived importance and self-evaluation emerged. While they recognized professional knowledge and learning methods as theoretically important (high WV), their self-evaluation scores (CEV) in these areas were the lowest among the three groups. Rather than a simple “reconciliation” with failure, this suggests a cognitive detachment where students acknowledge their deficiencies but no longer perceive them as addressable or emotionally distressing.

This is evidenced by their reported highest satisfaction with campus life and teaching quality, coupled with significantly lower levels of self-doubt compared to high-achievers. In other words, for this group, academic setbacks do not trigger significant emotional volatility. This pattern provides empirical substance to the “lying flat” (tang ping) mentality described in contemporary Chinese society (Zhang et al., 2025). Under the “strict-entry-and-lenient-exit” model, these students may develop a pragmatic confidence – relying on the institutional reputation of their alma mater for future employment rather than their individual academic mastery. Consequently, traditional interventions like notifications or standard counseling may prove ineffective, as the students’ cognitive maps have de-prioritized academic performance as a critical life-outcome driver.

Returning to our initial inquiry: Why do students at top universities experience exam failures? Our FCE analysis suggests that while deficiencies in professional knowledge are the direct cause, the underlying explanatory factor is the systemic weakening of the link between exam performance and future prospects. The findings indicate that for the struggling group, the absence of a direct linkage between failing a university exam and the inability to graduate or secure employment reduces the “cost” of failure. This suggests that the current examination system in high-level universities may require a thorough reconsideration of its purpose. Exams must not only serve as evaluation tools but also as meaningful checkpoints that students genuinely recognize as valuable for their professional development.

### 5.2 Limitation and future research

This study has several limitations that provide directions for future inquiry. First, our findings rely exclusively on self-perceived measures, which capture subjective psychological states but may be subject to individual reporting biases. Future research should aim to incorporate objective behavioral and institutional data to validate these self-assessments. Second, the single-institution sampling and the inherent cultural specificity of the Chinese elite university system limit the broader inference of our cognitive map interpretations. Comparative studies across different types of universities (e.g., vocational vs. research-intensive) and cross-cultural contexts (e.g., Confucian vs. Western) would enhance the robustness of the FCE framework. Finally, the cross-sectional nature of the current data means that the identified cognitive patterns should be viewed as distinct group profiles rather than confirmed developmental trajectories. Longitudinal tracking is recommended to explore how students’ cognitive-emotional configurations evolve over time in response to changing academic pressures.

## 6 Implication and conclusion

This study systematically investigated the cognitive, emotional, and behavioral disparities among university students with varying academic performance using the Fuzzy Comprehensive Evaluation (FCE) method. Our findings confirm that academic failure is not merely a lack of effort but a complex misalignment of cognitive maps. Specifically, we conclude that:

### 6.1 Cognitive disparities and agency

The FCE results demonstrate that high-achievers prioritize internal psychological drivers, whereas struggling students exhibit a detachment from personal agency, relying more on external institutional satisfaction.

### 6.2 The positive role of negative affect

In the Chinese high-level university context, negative affects like self-doubt and anxiety serve as significant positive drivers for success rather than inhibitors. This highlights the need for a culturally nuanced understanding of academic resilience.

### 6.3 Implication for university practice

Rather than direct policy mandates, these findings offer reflective insights for university administrators and faculty. First, support system should be tailored to specific cognitive profiles. For struggling students, the focus should shift from general counseling to rebuilding the “agency-performance” link, helping them re-internalize academic success as an achievable personal goal. Furthermore, the identified cognitive maps provide a psychological blueprint for the design of Open Educational Resources (OER). By adopting “OER-enabled learning” strategies, universities can provide personalized resources that specifically target the reconstruction of these agency-performance links, allowing struggling students to regain a sense of mastery through low-stakes, scaffolded digital engagement (Mishra, 2017). For fluctuating students, interventions should target environmental stabilizers to mitigate the impact of perceived external volatility. Second, these results invite a reconsideration of the core purpose of university exams. To move beyond a “lying flat” mentality, exams might be re-envisioned as meaningful milestones that provide clear, developmental value, rather than mere hurdles within a “lenient-exit” system. Finally, universities are encouraged to treat these proposed interventions as hypotheses for future testing. Future initiatives could employ small-scale pilot programs to empirically measure the impact of agency-focused training on the cognitive maps and subsequent academic trajectories of struggling students. University interventions should shift from improving external teaching facilities to rebuilding the internal self-efficacy and “agency-performance” links for struggling students.

## Data Availability

The raw data supporting the conclusions of this article will be made available by the authors, without undue reservation.
